# Dilated Cardiomyopathy in Pediatric Crohn’s Patient: Is It a Manifestation or Consequence of Therapy?

**DOI:** 10.7759/cureus.19357

**Published:** 2021-11-08

**Authors:** Fadi Busaleh, Hussain Albaqshi, Salsabeel AlSultan, Sarah Alateeq, Latifah A Alhashim, Zakariya Aldandan, Nawal Alfarhan

**Affiliations:** 1 Pediatric, Maternity and Children Hospital, Al-Ahsa, SAU; 2 Pediatrics, College of Medicine, King Faisal University, Al-Ahsa, SAU; 3 Pediatrics, Collage of Medicine, King Faisal University, Al-Ahsa, SAU; 4 Pediatric Gastroenterology, Maternity and Children Hospital, Al-Ahsa, SAU

**Keywords:** infliximab, biological therapy, inflammatory bowel disease, crohn’s disease (cd), ibd

## Abstract

Inflammatory bowel disease (IBD) is a debilitating chronic disorder that is classified into Crohn’s disease, ulcerative colitis, and unspecified which are marked by recurrent gastrointestinal inflammatory episodes. Anti-tumor necrosis agents, especially infliximab, are considered the cornerstone in disease management. However, rare but serious adverse effects related to infliximab have been reported. Limited studies reported cardiac adverse effects as a result of using infliximab in IBD especially in the pediatric age group. Here, we report a case of an 11-year-old boy known to have Crohn’s disease, who was on a regular infusion of infliximab at a monthly basis which developed dilated cardiomyopathy with severe depression of myocardial function.

## Introduction

Inflammatory bowel disease (IBD) is a debilitating chronic disorder that is classified into Crohn’s disease, ulcerative colitis, and unspecified which are marked by recurrent gastrointestinal (GI) inflammatory episodes. These chronic diseases are linked to higher morbidity as they have more frequent relapses and exacerbations that result in a lower quality of life [1]. Crohn’s disease and ulcerative colitis share similar characteristics with some differences based on the location of the affected bowel segment. IBD patients present with GI symptoms mainly diarrhea that results in malabsorption and failure to thrive. Despite this debilitating disease that has no cure, anti-tumor necrosis factor (anti-TNF) agents emerge as rescue medications that can be used for both induction and maintenance of remission of disease, especially with advanced cases [2]. Infliximab is one of the anti-TNF agents that has been used early with good outcomes. However, rare but serious complications related to this medication have been reported. Acute infusion reaction is an early adverse effect related mainly to infliximab, which presents with multiple signs and symptoms including chills, fever, sweating, headache, pruritus, flushing, dizziness, dyspnea, and reach up to anaphylaxis which is usually developed within 24 hours after drug infusion [3,4]. Moreover, it increases the risk of getting infections as it has an immunosuppressive effect, which includes reactivation of latent tuberculosis and chronic hepatitis B virus infection. Additional delayed complication associated with a higher dose is inducing malignancies such as lymphomas. Also, there are some associations between anti-TNF drugs and hepatic complications which range from asymptomatic elevation of liver enzymes to rarely acute liver failure. In addition, long-term infliximab administration causes autoantibodies formation which rarely leads to the lupus-like syndrome. Lastly, there is conflict about the anti-TNF effects on cardiac function. However, the New York Heart Association (NYHA) informs that anti-TNF drug is contraindicated for class III-IV heart failure [5]. Some cases of cardiomyopathy, acute heart failure, and sudden death are reported [6,7]. These cardiovascular complications are mainly reported in adults. The literature lacks such studies in pediatrics. Here, we report a case of an 11-year-old boy known to have Crohn’s disease, who was on a regular infusion of infliximab which developed dilated cardiomyopathy.

## Case presentation

An 11-year-old boy was diagnosed with Crohn’s disease at the age of nine years. Since then, he was on a regular infliximab transfusion regimen at monthly intervals at a dose of 5 mg/kg for maintenance of remission of disease as symptoms relapse by the end of each month. He presented to the Maternity and Children Hospital in Al-Ahsa, Eastern Province in Saudi Arabia, complaining of palpitation for one year. The palpitations were intermittent at the beginning of the year but then worsen progressively over the last month. They were associated with easy fatigability and chest discomfort, with no history of cyanosis or chest pain. There was no history of a similar condition or history of cardiac disease or sudden death in the family. In addition, these symptoms occur with the manifestation of tachycardia during infliximab transfusion with no respiratory or mucocutaneous involvement or other signs of anaphylaxis. This transfusion reaction is managed by slowing the transfusion rate and premedication with steroids and antihistamines.

**Figure 1:**
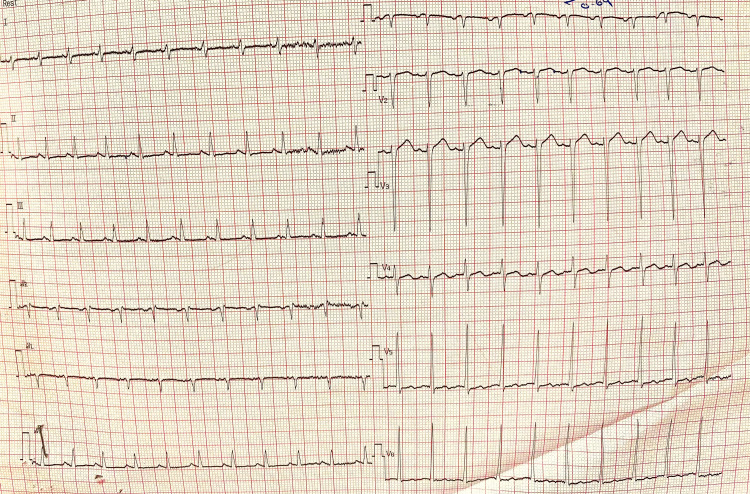
Twelve leads ECG showing sinus tachycardia (heart rate 150 beats/minute) with normal axis

**Figure 2:**
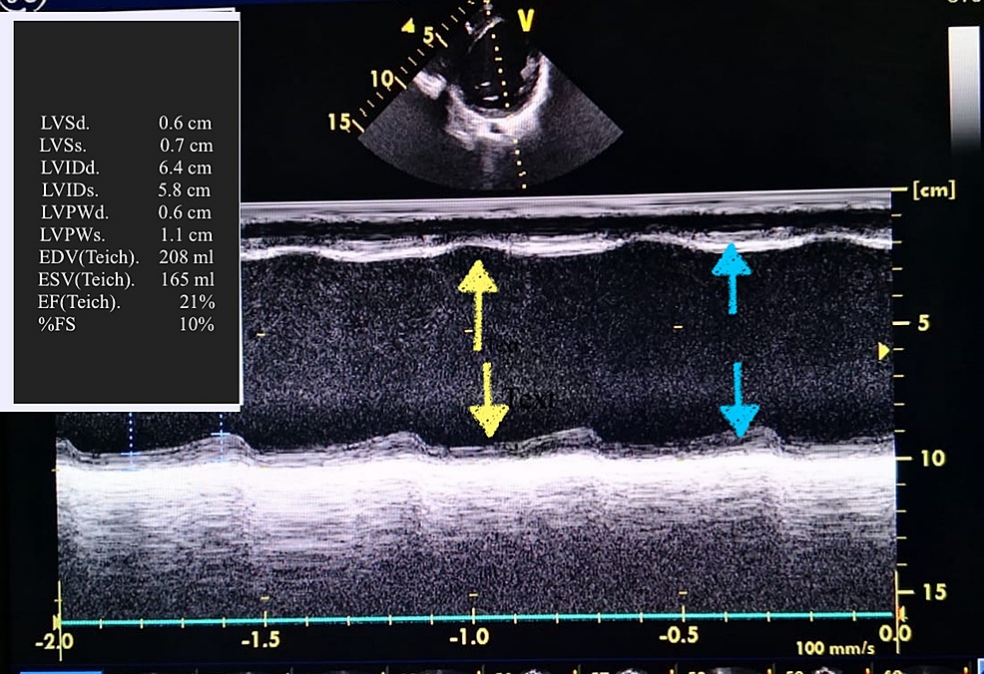
Echocardiogram M mode of left ventricle showing severe depression of myocardial contractility (blue arrows showing weak contractility) with wall asynchrony (yellow arrows showing incoordination between the wall muscles) and LV EDV 208 mL and LV ESV 165 mL with estimated ejection fraction of 21% and fraction of shorting of 10% LV EDV: left ventricular end-diastolic volume; LV ESV: left ventricular end-systolic volume; M mode: motion mode

Upon examination of the child, he appeared pale, underweight (with weight of 18 kg below the third centile) but not distressed. He had sinus tachycardia (150-160 beats/min) with maintained blood pressure (98/59 mmHg). chest examination revealed hyperdynamic precordium with pan-systolic murmur grade III out of VI at the apex with radiation to left mid-axillary line with no thrill. Rest of the examinations were unremarkable. Laboratory investigations showed microcytic hypochromic anemia related to the drop of iron profile and thrombocytosis, with positive anti-Saccharomyces cerevisiae antibodies for Crohn’s disease and negative antibodies for infliximab (Table 1). ECG and Holter 24 hours monitor were done and showed intermittent sinus tachycardia with no dysrhythmias (Figure 1). Echocardiography showed dilated left ventricle with ejection fraction of 21% and fraction of shorting of 10% associated with severe mitral regurgitation (Figure 2 and Video 1). The patient was diagnosed with acute heart failure secondary to dilated cardiomyopathy. He started on anti-failure medications controlling three parameters pre-load, after-load, and enhancing myocardial contractility by diuretics furosemide (1 mg/kg/dose twice a day initially), spironolactone (1 mg/kg/dose twice a day), enalapril (0.35 mg/kg/day divided three times a day) and digitoxin (2.5 mcg/kg/day once daily), respectively. Aspirin was added as an anticoagulant. In addition, the patient started on intravenous iron to improve anemia which was not responding to oral iron supplementations or dietary management. The patient was discharged home with the previous plan and kept on regular follow-up with pediatric cardiology with no need for cardiac surgery at the present time. In addition, he kept on regular follow up with pediatric hematology to assess the improvement in hemoglobin and response parental iron. Finally, the patient referred to a tertiary hospital for further follow-up with pediatric gastroenterology and the possibility of starting a different anti-tumor necrosis factor agent ustekinumab.

**Table 1:** Laboratory findings of the patient

Test	Result	Reference range
Complete blood count		
White blood cell count	7.98×10^3^	3-14×10^3 ^mg/dL
Hemoglobin	7.5	11.1-12.6 g/dL
Platelets	929×10^3^	150-350×10^3^
Mean corpuscular volume	50 fL	77-87 fL
Blood chemistry tests		
Serum sodium	132	133-152 mmol/L
Serum potassium	4	4.1-5.3 mmol/L
Serum calcium	2.02	2.12-2.52 mmol/L
Serum magnesium	0.95	0.74-0.99 mmol/L
Serum chloride	98	98-115 mmol/L
Serum phosphate	1.05	0.8-1.5 mmol/L
Blood glucose	96	74-106 mg/dL
Blood urea nitrogen	1.92	1.70-8.30 mmol/L
Creatinine	30.76	49-115 μmol/L
Iron level	2.7	reference 9-31.3 μmol/L
Total iron-binding capacity	19.28	44.75-80.55 μmol/L
Ferritin	286.6	13-150 ng/mL
Autoantibodies		
Anti-Saccharomyces cerevisiae antibodies IgG	68++	<20 RU/mL
Anti-Saccharomyces cerevisiae antibodies IgA	154+++	<20 RU/mL
Antibodies to infliximab	Negative	Negative

**Video 1:** Echocardiogram four chambers view showing severely dilated left ventricle with severe mitral regurgitation with maximum pressure gradient of 60 mmHg. Left atrium, right atrium and ventricle of normal size and shape with intact function. The tricuspid valve is normal with a pressure gradient of 12.9 mmHg

## Discussion

Biological therapy (immune therapy) is a new emerging modality in controlling debilitating inflammatory diseases through its antagonist action against the immune system that results in providing a good quality of life to the patient. Infliximab is an example of monoclonal antibodies that target the tumor necrosis factor which results in down-regulation of the immune system. It is considered the first biological therapy agreed by the Food and Drug Administration (FDA) in the United States for the treatment of inflammatory bowel diseases in pediatrics [3,5].

Despite the dramatic improvement associated with the use of infliximab some serious side effects can be encountered. FDA reported total cases of less than 100 patients, who developed new-onset heart failure or decompensation of preceded one and all were adults with no reported cases in pediatrics [3,5]. After that, infliximab was labeled to be contraindicated in cardiac patients with NYHA score III and beyond in adults. Our patient developed heart failure secondary to dilated cardiomyopathy, which we believed was the result of infliximab infusion as there were multiple occasions of palpitation and tachycardia concurrent with its infusion. It is baffling as some literature described that biological therapy in general, and infliximab in particular, can improve cardiomyopathy, but it is not conclusive [8]. In addition, usually, the reported cases are acutely seen, but here it was insidiously progressed, this may be related to the slower infusion rate adopted and the regular premedication with antihistamine and steroids [9]. The patient suffered from prolonged severe iron deficiency anemia which was related to both chronic blood loss from the gastrointestinal tract and the chronicity of the inflammatory disease which was resistant to standard treatment with oral iron supplementation but with parenteral one there was an improvement. Anemia by itself is a risk factor for the development of heart failure and can worsen a preexisting one [10]. Several kinds of autoantibodies are seen in Crohn's disease, and some are linked to the severity of the disease itself. Anti-Saccharomyces cerevisiae antibodies (ASCA) can be used for both a prognostic factor of Crohn's severity in addition to a differentiation bio-marker in between Crohn's disease (CD) and ulcerative colitis. In a study by Chandrakumar, there was a link between ASCA presence and severe gastrointestinal complications in Crohn's patients. This is concordant with our patient as he had a positive titer for ASCA and severe gastrointestinal manifestations that led to difficulty in controlling disease activity [11]. On the other hand, some literature suggest that dilated cardiomyopathy (DCM) is a part of CD and can be found either early, prior to treatment as in Oh et al., or later on, after treatment as in Bunu et al., but all agreed that most of the cardiovascular (CVS) complications are related to immune-related consequences. Unfortunately, no autoantibodies were linked to such a case [12,13].

Finally, palpitation started initially with the beginning of biological therapy, exactly during transfusion of infliximab, with no preceding of such event before. This possibly suggests that the anti-tumor necrosis factor (infliximab) is the triggering factor for dilated cardiomyopathy in our patient. On the other hand, negligence of Crohn's disease autoantibodies rules can't be excluded, especially that it had been linked before to cardiovascular complications in Crohn's disease [12,13].

## Conclusions

Biological therapy is considered the cornerstone in the management of Crohn's disease patients. Although the dramatic improvement that can be implanted through its usage can cause serious side effects. Dilated cardiomyopathy and subsequent heart failure are side effects of infliximab. At the same time, patients with Crohn's disease are susceptible to cardiovascular complications. So, regular follow up with cardiologists should be taken into consideration in managing Crohn’s disease patients.

## References

[1] Loftus EV Jr, Sandborn WJ (2002). Epidemiology of inflammatory bowel disease. Gastroenterol Clin North Am.

[2] Fakhoury M, Negrulj R, Mooranian A, Al-Salami H (2014). Inflammatory bowel disease: clinical aspects and treatments. J Inflamm Res.

[3] Siegel CA, Hur C, Korzenik JR, Gazelle GS, Sands BE (2006). Risks and benefits of infliximab for the treatment of Crohn's disease. Clin Gastroenterol Hepatol.

[4] Subedi S, Gong Y, Chen Y, Shi Y (2019). Infliximab and biosimilar infliximab in psoriasis: efficacy, loss of efficacy, and adverse events. Drug Des Devel Ther.

[5] Dogra S, Khullar G (2013). Tumor necrosis factor-α antagonists: side effects and their management. Indian J Dermatol Venereol Leprol.

[6] Emmert MY, Salzberg SP, Emmert LS (2009). Severe cardiomyopathy following treatment with the tumour necrosis factor-a inhibitor adalimumab for Crohn’s disease. Eur J Heart Fail.

[7] de’ Clari F, Salani I, Safwan E, Giannacco A (2002). Sudden death in a patient without heart failure after a single infusion of 200 mg infliximab: does TNF-alpha have protective effects on the failing heart, or does infliximab have direct harmful cardiovascular effects?. Circulation.

[8] Mera RE, Banch H, Torres EA (2007). Improvement of cardiomyopathy after infliximab treatment for Crohn's disease (CD). Am. J. Gastroenterol.

[9] Coletta AP, Clark AL, Banarjee P, Cleland JG (2002). Clinical trials update: renewal (renaissance and recover) and attach. Eur J Heart Fail.

[10] Puri K, Price JF, Spinner JA (2020). Iron deficiency is associated with adverse outcomes in pediatric heart failure. J Pediatr.

[11] Chandrakumar A, Georgy M, Agarwal P, 't Jong GW, El-Matary W (2019). Anti-Saccharomyces cerevisiae antibodies as a prognostic biomarker in children with Crohn Disease. J Pediatr Gastroenterol Nutr.

[12] Oh IS, Choi CH, Park JH (2012). A case of acute myocarditis as the initial presentation of Crohn's disease. Gut Liver.

[13] Bunu DM, Timofte CE, Ciocoiu M, Floria M, Tarniceriu CC, Barboi OB, Tanase DM (2019). Cardiovascular manifestations of inflammatory bowel disease: pathogenesis, diagnosis, and preventive strategies. Gastroenterol Res Pract.

